# Comparing effectiveness of two client follow-up approaches in sustaining the use of Long Acting Reversible Contraceptives (LARC) among the underserved in rural Punjab, Pakistan: a study protocol and participants’ profile

**DOI:** 10.1186/1742-4755-12-9

**Published:** 2015-03-18

**Authors:** Syed Khurram Azmat, Waqas Hameed, Moazzam Ali, Muhammad Ishaque, Ghulam Mustafa, Omar Farooq Khan, Ghazunfer Abbas, Erik Munroe

**Affiliations:** Department of Uro-gynecology, Ghent University, Sint-Pietersnieuwstraat 25, B-9000 Ghent, Belgium; Research, Monitoring & Evaluation Department, Marie Stopes Society, Karachi, Pakistan; Department of Reproductive Health and Research, World Health Organization, avenue Appia 20, 1211, Geneva 27, Switzerland; Research, Monitoring and Evaluation Department, Marie Stopes International, London, UK

**Keywords:** Family planning, Long acting reversible contraceptives, Method discontinuation, Non-inferiority prospective client follow-up, Maternal health, Study protocol, Punjab, Pakistan

## Abstract

**Background:**

Pakistan observes a very high i.e. 37 percent modern contraceptive method related discontinuation rates within 12 months of their initiation. And almost 10 percent of these episodes of discontinuation happened due to the side effects or health concerns experienced by the women. Most importantly, it was noted that more than 12,000 first-level care facilities are located in the rural areas, including rural health centers, basic health units, and family welfare centers, but more than 30% of these facilities are nonfunctional. This paper presents a study protocol and participants’ profiling of a prospective cohort follow-up to compare the effectiveness of household based and telephonic approaches in sustaining the use of Long Acting Reversible Contraceptives (LARC) whilst to facilitate lowering method related discontinuation and increasing switching amongst the contraceptive users.

**Methods:**

A 12-month multi-centre, non-inferiority prospective user follow-up is employed using three different study categories: a) household based follow-up; b) telephonic follow-up; and c) passive or need-based follow-up along with the hypothetical assumption that the telephonic client follow-up is not inferior to the household based follow-up by continuation rate of LARC and the telephonic follow-up is less-costly than the household based client follow-up. This follow-up will be conducted in 22 health facilities – (16 rural and 6 urban based facilities) in district Chakwal. The first two study categories will receive scheduled but different follow-up from the field workers at 1, 3, 6, 9, and 12 month while the third one i.e. the ‘passive or need-based follow-up’ will serve as a control group. Using sampling software PASS 11, it was estimated to have 414 clients in each study category and around 1366 clients will be recruited to account for 10% attrition rate.

**Discussion:**

The study will help us to examine a more convenient method of effective follow-up for managing side effects, decreasing method discontinuation and increasing switching amongst users. The study information will also facilitate to develop a robust, effective and efficient mechanism for client follow-up to promote the continuation rates of LARC methods. The follow-up results and lessons learnt will be widely shared with stakeholders for their implementation and streamlining in health system.

## Background and context

Family planning (FP) is considered as one of the most cost effective interventions and has an imperative function to reduce maternal, infant and child health related morbidity and mortalities - as such that it has the potential to prevent around 30% of maternal deaths and 10% of child deaths [1, 2]. But the benefits of family planning are not limited just to promoting maternal or child health but can significantly influence enhanced opportunities for higher socioeconomic status, education, employment and empowerment especially for girls and women [3]. Likewise, family planning/contraception use also reduces the rates of unskilled unsafe abortions by preventing the mistimed or unwanted pregnancies [2].

But unfortunately, almost 300,000 women died only in 2013 due to pregnancy related complications which were avoidable [4, 5] and the preventable causes such as hemorrhage, hypertension and infections accounted for 56.8% of all maternal deaths [6]. It was noted that in the absence of contraceptive use, these deaths are projected to be almost two-fold higher [7].

In Pakistan, the lifetime risk of maternal death is one in 170 for women [8], and Pakistan is one of the six countries in the world anticipated to contribute to over half of all maternal deaths and complications of pregnancy and childbirth remain the leading cause of death and disability for childbearing women in Pakistan [9].

During the past 22 years, there is a three-fold progressive but gradual increase in the contraceptive use in Pakistan i.e. from 12 percent in 1990–91 to 30 percent in 2006–07 and then to 35 percent in 2012-13 [10, 11]. Likewise, the unmet need for family planning decreased from 31 percent to 20 percent during this period. But very unfortunately, modern contraceptive use remain as low as at 26 percent in contrast to the extremely high and favorable universal knowledge of contraception (almost 99%) with more dependency on short term, traditional and permanent methods of contraception [10]. Most importantly, the use of highly effective long acting reversible contraceptives (LARC) such as IUD and implant has remained almost negligible for a decade [1, 12].

The country also observes high contraceptive discontinuation rates of 37 percent within 12 months of their initiation resulting in particular from the side effects or health concerns associated mainly with short term modern contraceptives such as injections, pills and condoms [10].

In Pakistan, in a recent retrospective study in 2011 the contraceptive users revealed high IUD discontinuation among users (16.3% at 12 months) [13] while the implant 1-year discontinuation stands at 10% [14]. Importantly, the recent demographic survey reported a very high 26% of IUD discontinuation rate at 12 month. However, the main reason cited for IUD discontinuation was side effects in almost all the cases [10, 13, 14].

To promote modern family planning methods especially LARC methods, Marie Stopes Society (MSS), a local non-governmental organization in Pakistan, implemented a research project titled “Meeting the birth spacing needs of the underserved in Punjab province in Pakistan” and used demand-side financing – (DSF) through vouchers and expanding the *Suraj* health providers model (a Social Franchise (SF) network of health providers) as a two-pronged approach in order to increase the use and uptake of modern contraceptives [15]. Earlier evidence suggests that the DSF vouchers and social franchising; as an integrated approach used for LARC choices, has the potential to increase awareness and use of LARC methods [16, 17].

In the MSS Pakistan implemented research project titled “Meeting the birth spacing needs of the underserved in Punjab Province in Pakistan” the Field Health Educators (FHE) are working as a Community Health Worker within the *Suraj* model and has a similar role to a Lady Health Worker – LHW (LHW Programme is a Government of Pakistan owned Primary Health Care Initiative) and covers a population of around 20,000 to 25,000 spread over 3 – 6 kilometer radius [15]. This population catchment is far higher compared with the population and catchment areas of LHWs and Community Midwives – CMWs (CMW programme is a Maternal, Newborn and Child Health Initiative of the Government of Pakistan mainly for the population residing in the most far-flung rural areas) [15]. Each LHW serves a population of 1,000 people in their rural community while one CMW stationed for 5000 - 10,000 populations [15, 18–20]. This makes it challenging for FHE to frequently pay household based follow-up visits to the clients.

A large number of systematic reviews have documented the important role of community-based interventions including the household-based visits by community health workers to improve maternal and newborn health outcomes [21]. Likewise, several studies have cited that by strengthening the counseling and follow-up services component of the family planning programs will support women’s continuation of effective methods and ensure higher quality of services [22–24]. For example, a pool of studies have attempted to measure the effects of different approaches of user follow-up such as an scheduled visit at the clinic or a follow-up telephone contact and have reported mixed results; however, these studies has design and quality limitations [25]. Another study identified the importance of follow-up at six weeks for detecting early expulsion but the same study did not found any significant association between the regular visits and fewer pregnancies or expulsions [26]. Amongst those who cited medical reasons for the removal of IUD, there was a significant difference between the more frequent follow-up users as compared to the fewer scheduled follow-up as the earlier had a longer period of IUD continuation [26]. However, effect of a follow-up telephone call was not found as significantly different [27–30]. Unfortunately, sparse evidence is available on utility of household or telephone based client follow-up for family planning in Pakistan and in the neighboring countries.

Thus, there is a need to study effects of prospective follow-up to the end-user and counseling related to side-effect management by a provider or community health worker in the rural setting in Pakistan. Therefore, we propose to conduct a prospective cohort follow-up to compare the effectiveness of household based and telephonic approaches in sustaining the use of LARC among SF clients. Very recently, the Department of Reproductive Health and Research at the World Health Organization in Geneva unveiled the 15 highest priority topics as their global agenda to promote family planning research [2]. Amongst those identifying main barriers to the uptake and use of modern contraceptive in low prevalence settings and the determinants of the discontinuation as well as switching of contraceptive methods seem extremely relevant and timely for our study [2]. In addition, engagement with the private sector through franchising; task shifting and impact of financing schemes on quality are also pertinent for our study [2].

Following are research questions, hypothesis and list of measurement indicators of the proposed study:

**Research Question**

1. Is telephonic client follow-up on LARC continuation is as effective as the effect of (gold standard) active household based client follow-up?
2. Is the telephonic client follow-up less costly compared with the household based client follow-up?

**Hypothesis**

1. The telephonic client follow-up is not inferior to the household based follow-up by continuation rate of LARC.
2. The telephonic client follow-up is less-costly than the household client follow-up.

**List of follow-up measurement indicators**

1. The cumulative probability of LARC discontinuation 1, 3, 6, 9, and 12 months by study arms.
2. The reasons for LARC discontinuation
3. Level of client satisfaction with the counseling received from FHE by study arms
4. The proportion of clients lost to follow-up
5. Cost per telephonic call and per household based visit
6. Time duration per call
7. Level of difficulty faced by client in contacting FHE by follow-up group

## Methodology

The present study is nested in the larger research project - Meeting the birth spacing needs of the underserved in Punjab Province in Pakistan [15].

### Design

A multi-centre, prospective non-inferiority trial.

### Scope

This 12 month follow-up from December 2013 – December 2014 will be conducted only in Chakwal district nested under the routine monitoring of programme activities on Demand Side Financing [15]. A total of 22 centers will be included in this follow-up, of which 16 are mid-level providers branded as Suraj (i.e. in English, called Sun, is a brand provided to the clinics of the trained providers of MSS) and located in rural periphery, while the 6 are medical doctors based in peri-urban or urban areas.

### Follow-up cohorts

The recruitment of follow-up participants will take place under three different categories: a) household based follow-up; b) telephonic follow-up; and c) passive or need-based follow-up, serving as control group. The clients recruited under category ‘a’ will receive a scheduled follow-up from FHE at 1, 3, 6, 9, and 12 month. Similarly, the clients recruited under category ‘b’ will receive a telephone call from FHE at the scheduled frequency similar to category ‘a’. In addition, the participants of both the categories (‘a’ & ‘b’) can also seek medical advice from FHE based on need (unscheduled contact). While, category ‘c’ participants will not receive any scheduled intervention from FHE but can only seek FHE’s assistance in case of any medical need.

### Sample size

In order to detect 6% difference amongst the categories, with an 80% power and using sampling software PASS 11, it was estimated to have 414 clients in each of the three groups. Around 1366 clients will be recruited to account for 10% attrition rate. Based on services statistics the estimated sample can be achieved if all the clients at each clinic (who gives informed consent) are registered during the (specified) period for 3-4 months. Thereby clinics having high clientele will contribute more to the sample (to hold proportionality assumption).

### Sampling strategy

Based on preliminary data taken from the field, it is estimated that around 40% of the LARC users have mobile phones [31] thus due to shorter recruitment time, selection of follow-up participants will be done following consecutive sampling. The reason for selecting consecutive sampling over randomization was mainly the long recruitment periods and the budgetary implications. After screening, all the clients receiving LARC during 3-4 months (Nov 2013 till Feb 2014) period will be briefed about follow-up and invited to participate in the follow-up based on their informed consent. Clients will be asked to voluntarily choose the group (among the three described above) under which she would like to be enrolled. Once the desired sample of any specific group is achieved, that group will be eliminated as an option for subsequent clients to opt for. A format will be given to each service provider to keep a track of client enrollment under each group. The same information shall be recorded on the baseline and follow-up form so as to ascertain the selection is performed as per the protocol.

### Tool

A structured questionnaire at baseline and 12-month will be administered to document socio-demographic characteristics and user-related reasons (decision making, acceptability, costs, convenience of method type, timing) and health-related reasons reported by the women. The questionnaire was developed by MSS and reviewed by Department of Reproductive Health and Research, WHO. Moreover, all the enumerators will be given thorough training on all the questionnaires.

### Implementation

Once the client adopted the LARC, she will be asked to participate in the follow-up. As described above, the women enrolled under the active categories (‘a’ & ‘b’) will receive counseling on side-effect management, through an adapted manual used in the Family Advancement for Life and Health (FALAH) project [32] at 1, 3, 6, 9 and 12-month post IUCD insertion. The FALAH was a family planning project supported by the United States Agency for International Development (USAID) whose aim was to protect the health and well being of mothers, newborns and children through the adoption of birth spacing by eligible couples in Pakistan [33]. The same manual will be used to assist women with side-effect management as per need. In order to minimize dropouts/lost-to-follow-up, all the clients will be informed about the detailed follow-up procedures, while taking informed consent. For schedule visits, the FHEs will be reimbursed for their transportation cost. Similarly, FHEs will also be paid for telephone call expenses.

## Baseline information

### Socio-demographic characteristics and participants’ profile

The socioeconomic profile had N = 424, 409 and 428 women for the Telephone, Physical and Passive follow-up categories respectively (refer to Table 1). All three categories showed the largest number of women in the age group of 24 to 35 years of age. Most of the women in all of the categories were of twenty years of age or less at the time of their marriage and the average age of husbands for majority was between 30 and 40 years range for all categories.

**Table 1:** Socio-demographic characteristics and participant’s profile by prospective client follow-up group*

Indicators	Telephone group	Physical group	Passive group
	N = 424	N = 409	N = 428
	%	%	%
**Age of women**			
15 – ≤ 24	10.4	7.8	8.2
>24 – ≤ 34	66.7	63.3	60.5
≥35	22.9	28.9	31.3
Average (S.D)	30.4 (4.7)	31.0 (4.9)	31.0 (5.2)
***P-value***	***0.06***		
**Age of women at time of marriage**			
≤20	62.0	63.8	62.6
>20 – ≤ 25	31.1	28.9	29.0
>25	6.8	7.3	8.4
Average (S.D)	20.3 (3.1)	20.3 (3.1)	20.6 (3.2)
***P-value***	***0.86***		
**Age of husband**			
≤30	23.1	17.4	20.6
>30 – < 40	46.7	47.2	41.6
≥40	30.2	35.5	37.9
***P-value***	***0.06***		
**Women education**			
No formal education	36.3	40.8	43.2
Can read/write	4.7	3.2	2.6
Primary	12.0	15.9	13.1
Middle	11.1	15.6	12.4
Secondary	25.7	17.4	19.4
Intermediate	6.1	4.9	5.1
Graduate/Post Graduate	4.0	2.2	4.2
***P-value***	0.03*		
**Working women**			
Yes	9.7	8.6	6.8
Housewife	90.3	91.4	93.2
***P-value***	***0.30***		
**Husband education**			
No formal education	15.6	15.2	20.1
Can read/write	2.4	2.9	0.5
Primary	7.8	10.8	9.6
Middle	19.1	19.6	17.1
Secondary	42.2	42.8	43.9
Intermediate	8.5	6.4	4.4
Graduate/Post Graduate	4.5	2.4	4.4
***P-value***	0.03*		
**Husband occupation**			
Unskilled manual	18.4	21.8	22.9
Skilled manual	21.9	21.5	21.7
Agriculture/Farming	11.3	16.6	17.3
Sales & services	17.0	14.9	13.8
Professional/Technical manager	17.5	12.7	13.3
Clerical/Office work	7.5	5.9	5.1
Household chores	2.6	1.5	1.9
Jobless	3.8	5.1	4.0
***P-value***	***0.20***		
**Family monthly income (PKR) (1 USD = 102 PKR)** ^**1**^			
≤5,000 (49 USD)	13.6	13.8	14.2
>5,000 – ≤10,000 (>49 USD – 98)	55.1	58.8	52.8
>10,000 – ≤15,000 (>98 USD – 147)	22.9	20.0	19.3
>15,000 (>147 USD)	8.4	7.4	13.6
Average (S.D)	10,282 (7654)	10,263 (9,149)	10,433 (5,166)
	100.8 USD (75)	100.6 (89.7)	102.3 (50.7)
Median	9,000	9,000	9,000
	88.2 USD	88.2 USD	88.2 USD
***P-value***	***0.12***		
**Type of client**			
Out of Pocket (referred by FHE)	5.0	9.8	8.4
Voucher	95.0	90.2	91.6
***P-value***	0.03*		
**Type of Provider**			
*Suraj* SF (Rural)	67.0	68.7	69.6
Urban Provider (Urban)	33.0	31.3	30.4
***P-value***	***0.70***		
**FP method taken**			
IUD	78.5	85.3	85.0
Implant	21.5	14.7	15.0
***P-value***	0.01*		

*Groups: Telephone group will receive a telephone call at the scheduled frequency; Physical group will receive household based follow-up, and Passive group category participants will not receive any scheduled intervention but can only seek assistance in case of any medical need.

^1^ http://www.forex.pk/open_market_rates.asp.

Majority of the women in all three categories had no formal education (observed as 36.3%, 40.8% and 43.2% for Telephone, Physical and Passive categories respectively).

Moreover the ‘type of client’ indicator showed that majority i.e. Telephone (95%), Physical (90.2%) and Passive (91.6%) obtained their FP services via vouchers. The percentage for IUCD uptake was 78.5%, 85.3% and 85% for Telephone, Physical and Passive categories respectively.

The majority of all three categories showed a favorable view of the husband with regards to using a contraceptive method (refer to Table 2).

**Table 2:** **Family views regarding FP contraceptive by prospective client follow-up group**

Indicators	Telephone group	Physical group	Passive group
	N = 424	N = 409	N = 428
	%	%	%
**Husband’s view on couple using a contraceptive method**			
Favors	81.7	71.8	74.3
Dislikes	8.3	15.9	13.9
Don’t know/unsure	10.0	12.3	11.8
***P-value***	***<0.01***		
**Husband knows you have inserted IUCD/Implant**			
Yes	83.3	76.3	72.2
No	16.7	23.7	27.8
***P-value***	***<0.001***		
**Mother in law knows you have inserted IUCD/Implant**			
Yes	51.4	46.5	40.7
No	48.6	53.5	59.3
***P-value***	***<0.01***		

### Reproductive health and contraceptive history

Reproductive health and contraceptive history show that most of the women in each group had 3-4 pregnancies and also had similar number of living children as well (Refer to Table 3).

**Table 3:** **Reproductive health and contraceptive history by prospective client follow-up group**

Indicators	Telephone group	Physical group	Passive group
	N = 424	N = 409	N = 428
	%	%	%
**Number of pregnancies**			
1 – 2	32.5	27.4	27.8
3 – 4	44.1	45.7	47.0
5 or more	23.3	26.9	25.2
Average (SD)	3.5 (1.8)	3.7 (1.8)	3.6 (1.8)
***P-value***	***0.43***		
**Number of living children**			
0 – 2	38.0	32.0	34.1
3 – 4	44.8	49.4	49.8
5 or more	17.2	18.6	16.1
Average (S.D)	3.1 (1.6)	3.3 (1.5)	3.2 (1.5)
***P-value***	***0.37***		
**Result of last pregnancy**			
Live birth	93.9	91.9	90.0
Still birth	3.8	5.1	5.4
Miscarriage/aborted	2.4	2.9	4.7
***P-value***	***0.25***		

The percentage of women who did not use any contraception in the past three months was 44% in each Telephone, Physical and Passive categories (Table 3). When questioned about ‘spacing’ with regards to the ‘desire to have more children’; many women expressed for a desired spacing period of 36 months before their next child.

### Information pertaining to IUCD/Implant services

Long acting effectiveness was the main reason for choosing IUCD/Implant among Telephone (61.3%), Physical (59.2%) and Passive (55.3%) categories. Similarly the decision to take up the service was made mostly by the women themselves in all three categories; while mutual decision by husband and wife was identified as second most common factor (refer to Table 4). It was noted that the majority of the participants (more than 75%) reported having no side effects during ICUD/implant insertion amongst all categories. Likewise, most of the women (more than 65%) didn’t get any painkillers to take home after the insertions.

**Table 4:** **Reason and final decision making regarding insertion of IUCD/Implant by prospective client follow-up group**

Indicators	Telephone group	Physical group	Passive group
	N = 424	N = 409	N = 428
	%	%	%
**Reason for choosing IUCD/Implant**			
Suggested by FHE (Suraj)	7.9	8.3	9.6
Suggested by FHE (Urban)	7.4	8.8	8.2
Suggested by Suraj client	4.1	4.6	1.6
Long acting effectiveness	61.3	59.2	55.3
Freely available	7.2	7.6	7.7
Easily accessible	8.6	7.3	13.1
Others (fewer side effects, suggested by husband/LHW/Urban happy client)	3.5	4.1	4.4
***P-value***	***0.14***		
**Final decision made by**			
Myself	57.5	62.3	64.6
Husband	1.4	0.0	0.0
Both (husband & wife)	36.3	32.8	32.8
Mother in law	0.9	0.7	0.5
All together	3.8	1.9	1.9
***P-value***	***0.08***		
**Experienced side effects during IUCD/Implant insertion**			
Yes	21.9	24.4	21.5
No	78.1	75.6	78.5
***P-value***	***0.71***		
**Got the painkillers to take home**			
Yes	32.9	38.4	32.6
No	67.1	61.7	67.4
***P-value***	***0.35***		

### Counseling and quality of information provided

The majority of the clients reported that they received counseling on all family planning methods before they opted for IUD or Implant (refer to Table 5). As shown in Table 5, most of the women in Telephone (89%), Physical (91%) and Passive follow up (92%) categories got clear instructions in case they experience any side effects. Similarly, majority of the women among all categories got the method of their choice.

**Table 5:** **Receive counseling on FP method before IUCD/Implant insertion by prospective client follow-up group**

Indicators	Telephone group	Physical group	Passive group
	N = 424	N = 409	N = 428
	%	%	%
**Contraceptive method (multiple responses)**			
None/Didn’t discuss any method	6.1	11.0	7.7
***P-Value***	***0.03***		
Condoms	80.7	75.1	74.8
***P-Value***	***0.07***		
Pills	84.0	78.7	80.6
***P-Value***	***0.15***		
Injections	85.8	80.0	84.3
***P-Value***	***0.06***		
IUCD	89.6	83.6	87.9
***P-Value***	***0.03***		
Implant	72.2	70.9	70.1
***P-Value***	***0.80***		
Female sterilization	17.0	23.0	19.4
***P-Value***	***0.09***		
Male sterilization	9.4	14.4	10.3
***P-Value***	***0.05***		
Withdrawal	4.5	5.9	5.6
***P-Value***	***0.64***		
Periodic abstinence	7.3	9.3	8.9
***P-Value***	***0.55***		
Emergency contraception	8.5	7.8	6.5
***P-Value***	***0.55***		

## Discussion

Based on the results of the study, we hope to identify a more convenient method of effective follow-up for managing side effects, health concerns and other potential reasons of method discontinuation and switching. The study information will also facilitate to develop a robust, effective and efficient mechanism for client follow-up in Suraj Social Franchise model for FHEs to promote the continuation rates of LARC methods. The follow-up results and lessons learnt will be widely shared with stakeholders (both amongst government and private sector) for their replication in health system. We will also use the follow-up findings to contribute to the knowledge base through publications in peer reviewed journals, working paper series and relevant international/regional conferences.

Depending on the results it is anticipated that we will have relatively efficient, easy and less tiring (less mobility) approach to ensure post-method uptake follow-up. This will also provide supportive environment in managing post-insertion follow-up for LARC users in the rural areas in order to reduce higher method discontinuation and switching. We hope study findings will help large cadre of LHWs and other outreach workers to effectively follow-up clients and eventually leading to reduction in contraceptive discontinuation in the country.

## Technical support

The role of the Department of Reproductive Health and Research (RHR) at WHO is to provide technical advisory support to the in-country project team/Principal Investigator (PI). This is not just limited to supervising the Monitoring and Evaluation (M&E) aspect of the study, but also to review and share continuous feedback on the project activities and to create an enabling environment for informed decision-making on operational issues, research and policy or strategy. Moreover, multiple peer-reviewed publications in professional international journals, best practices, case studies, conference presentations and policy briefs will be produced through a consultative process that involves the RHR technical adviser and the study PI/project team.

## Ethical considerations

The study protocol of the research project has received approval by Research Ethics Committee of the National Bioethics Committee (Ref: No. 4-87/12/NBC-92/RDC/3548) and it is already published in the journal *Reproductive Health* (http://www.reproductive-health-journal.com/content/11/1/39) [15, 31]. Written informed consent will be taken from all survey participants in a manner that is appropriate for the local language, literacy level and comprehension level of the target population. Every respondent, irrespective of whether information is being elicited using quantitative methods, will be informed about the nature and objectives of the project, any real or perceived benefits or risks, and assurances of confidentiality. All respondents have the right to refuse to answer any question posed by the interviewer, and that s/he may also stop the interview at any time and there are no financial incentives for the participation in the study. Moreover, no personal identifying information will be recorded on questionnaires that can be used to link recorded information to a specific individual. Steps will also be taken to ensure privacy during interviews.

## Potential risks

There will be no potential risks to the participants whilst participating in the study except some loss of confidentiality which might occur during the study. To minimize this, training will be provided to the study staff to ensure and minimize breach of confidentiality of participants. Participants’ contact information and informed consent documents with participants’ names will be stored separately in lockers. No personal identifiers for participants will appear on any data or documentation sent to the funding organization. Participants will not be identified by name in any report or publication resulting from the study data.

## Limitations

The follow-up will assess early discontinuation up to 12 months. There may be likely chance of some recall bias in exact date/timing of discontinuation as the data will be collected immediately after the last (12-month) follow-up visit. Providers’ bias is also expected for specific FP methods, furthermore due to non-randomization, clients’ observed and unobserved characteristics may confound the results.
